# Long-range structural preformation in yes-associated protein precedes encounter complex formation with TEAD

**DOI:** 10.1016/j.isci.2022.104099

**Published:** 2022-03-17

**Authors:** Michael Feichtinger, Andreas Beier, Mario Migotti, Matthias Schmid, Fedir Bokhovchuk, Patrick Chène, Robert Konrat

**Affiliations:** 1 Department of Computational and Structural Biology, Max Perutz Labs, University of Vienna, Campus Vienna Biocenter 5, 1030 Vienna, Austria; 2 Ichnos Sciences SA, Route de la Corniche 5A, 1066 Epalinges, Switzerland; 3 Novartis Pharma AG, Postfach WSJ 386.4, 4002 Basel, Switzerland

**Keywords:** Biochemistry, Cell biology, Structural biology

## Abstract

Yes-associated protein (YAP) is a partly intrinsically disordered protein (IDP) that plays a major role as the downstream element of the Hippo pathway. Although the structures of the complex between TEA domain transcription factors (TEADs) and the TEAD-binding domain of YAP are already well characterized, its apo state and the binding mechanism with TEADs are still not clearly defined. Here we characterize via a combination of different NMR approaches with site-directed mutagenesis and affinity measurements the intrinsically disordered solution state of apo YAP. Our results provide evidence that the apo state of YAP adopts several compact conformations that may facilitate the formation of the YAP:TEAD complex. The interplay between local secondary structure element preformation and long-range co-stabilization of these structured elements precedes the encounter complex formation with TEAD and we, therefore, propose that TEAD binding proceeds largely via conformational selection of the preformed compact substates displaying at least nanosecond lifetimes.

## Highlights

• Secondary structure elements are preformed in apo YAP
• Preformation of secondary structure elements is co-dependent
• Apo YAP exhibits long-range structural compaction
• YAP compaction has a kinetic contribution to the YAP:TEAD formation

## Graphical abstract

**Figure:**
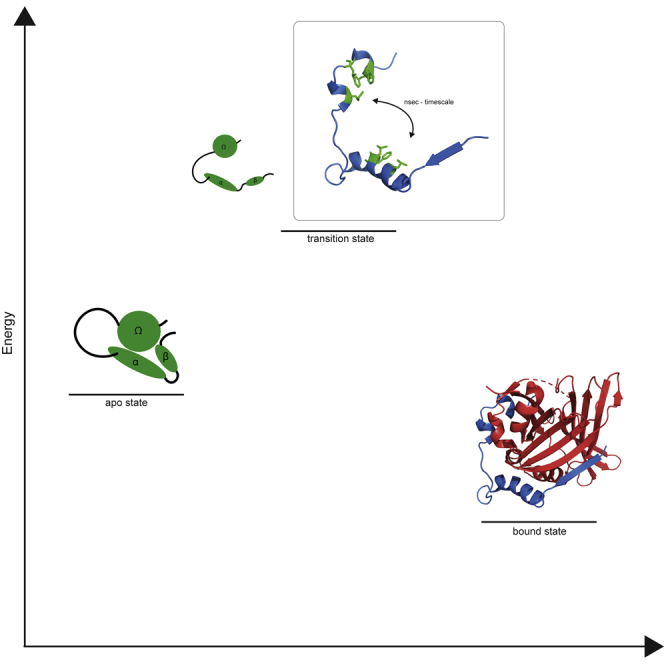

## Introduction

YAP (Yes-Associated Protein) is a partly intrinsically disordered protein (IDP) that serves as one of the terminal effectors of the Hippo pathway. This pathway plays a central role in regulating organ size, cell differentiation, and regeneration (Moya and Halder, 2019; Tremblay and Camargo, 2012; Wang et al., 2017; Zhao et al., 2011). One of the main functions of YAP is to bind the TEA/ATTS domain transcription factors (TEADs), as a transcriptional co-activator. Together, YAP and TEAD form a functional transcription complex that induces the expression of Hippo-responsive genes (Pobbati et al., 2015; Pobbati and Hong, 2013). The formation of the YAP:TEAD complex is regulated by YAP phosphorylation on S127, which prohibits the nuclear translocation of YAP via 14-3-3 binding and leads to the downregulation of genes related to proliferation, therefore, leading to apoptosis (Zhao et al., 2007). The phosphorylation of YAP is a result of the activation of the core kinase cassette of the Hippo pathway and occurs in the cytoplasm (Ma et al., 2019). Thus, phosphorylation at S127 leads to cytoplasmic retention via binding to 14-3-3 proteins (Zhao et al., 2007). The deregulation of the Hippo pathway is associated with cancer development (Liu et al., 2012; Ma et al., 2015; Pobbati et al., 2015; Pobbati and Hong, 2013) and, therefore, the inhibition of the YAP:TEAD interaction might be a promising therapeutic approach in cancers where the Hippo pathway is deregulated (Calses et al., 2019; Holden and Cunningham, 2018; Santucci et al., 2015; Ye and Eisinger-Mathason, 2016).

The TEAD binding domain of YAP was mapped to the protein region 50-100 (Vassilev et al., 2001), and several crystal structures of YAP:TEAD complexes have been solved in recent years (Chen et al., 2010; Li et al., 2010; Mesrouze et al., 2017). These structures revealed three binding interfaces between YAP and TEAD: a β-strand (residues 52-58), an α-helix (residues 61-74), and an Ω-loop (residues 84-99). These three secondary structure elements bind to distinct regions at the surface of TEAD. It was shown that the mutation of certain residues in the α-helix and Ω-loop strongly affects binding affinities which lead to the conclusion that these two sites form the crucial binding interfaces in the YAP:TEAD interaction (Mesrouze et al., 2017). In addition, previous studies demonstrated that the presence of the α-helix and Ω-loop are necessary for binding with nanomolar affinity (Hau et al., 2013). Furthermore, key residues of YAP and TEAD that constitute the YAP:TEAD binding site and their individual contribution to the binding have been described by site-directed mutagenesis studies (Mesrouze et al., 2017). They revealed that crucial residues for the interaction of the α-helix are L65, L68, and F69 which belong to the LxxLF motif that is known to bind to hydrophobic sites (Li et al., 2010; Santucci et al., 2015). In a similar manner, the hydrophobic residues M86, L91, and F95 form a hydrophobic core within the Ω-loop region that interacts with TEAD. This core region is further stabilized by F96 through hydrophobic interactions. In addition, residues R89 and S94 interact via hydrogen bonds with TEAD. The mutations of these residues to Ala showed significant effects (ΔΔG from 1.9 to 4.4 kcal/mol) on the binding affinity between YAP and TEAD (Mesrouze et al., 2017).

While the complex between the YAP-binding domain of TEAD and the TEAD-binding domain of YAP is already well characterized, there are hardly any data on the apo state of the TEAD-binding domain of YAP that is defined as intrinsically disordered protein in the apo state (Feichtinger et al., 2018; Tian et al., 2010).

As already indicated, the YAP:TEAD interaction might be a promising therapeutic approach in cancers where the Hippo pathway is deregulated. However, there are some reports in the area of small molecules targeting the YAP:TEAD transcriptional activation complex with moderate levels of pathway inhibition (Crawford et al., 2018). Interestingly, it has been shown that Verteporfin, known to disrupt the YAP:TEAD interaction, is co-eluting with YAP and not with TEAD on a size exclusion column, and therefore, suggested to bind to YAP (Liu-chittenden et al., 2012). Even if further research has shown that porphyrin- and dipyrrin-related derivatives can directly target YAP (Gibault et al., 2017), the mechanism behind the inhibition effect of Verteporfin and these derivatives remains to be elucidated as there is no structural information on the apo state of YAP. In summary, all the studies carried out so far have mainly focused on the bound state of YAP to TEAD, mainly owing to the insufficiency of the applied methods for characterizing the intrinsically disordered YAP protein in the free form on an atomic level. The predominating opinion in the field is that the TEAD-binding domain of YAP is completely disordered and folds upon binding to TEAD.

In this article, we combine different NMR approaches with site-directed mutagenesis and affinity measurements to characterize the intrinsically disordered state of YAP. We present evidence that the apo state of YAP adopts several ordered conformations that may facilitate the formation of the YAP:TEAD complex.

## Results

### Secondary structure elements are preformed in apo YAP

**Figure 1:**
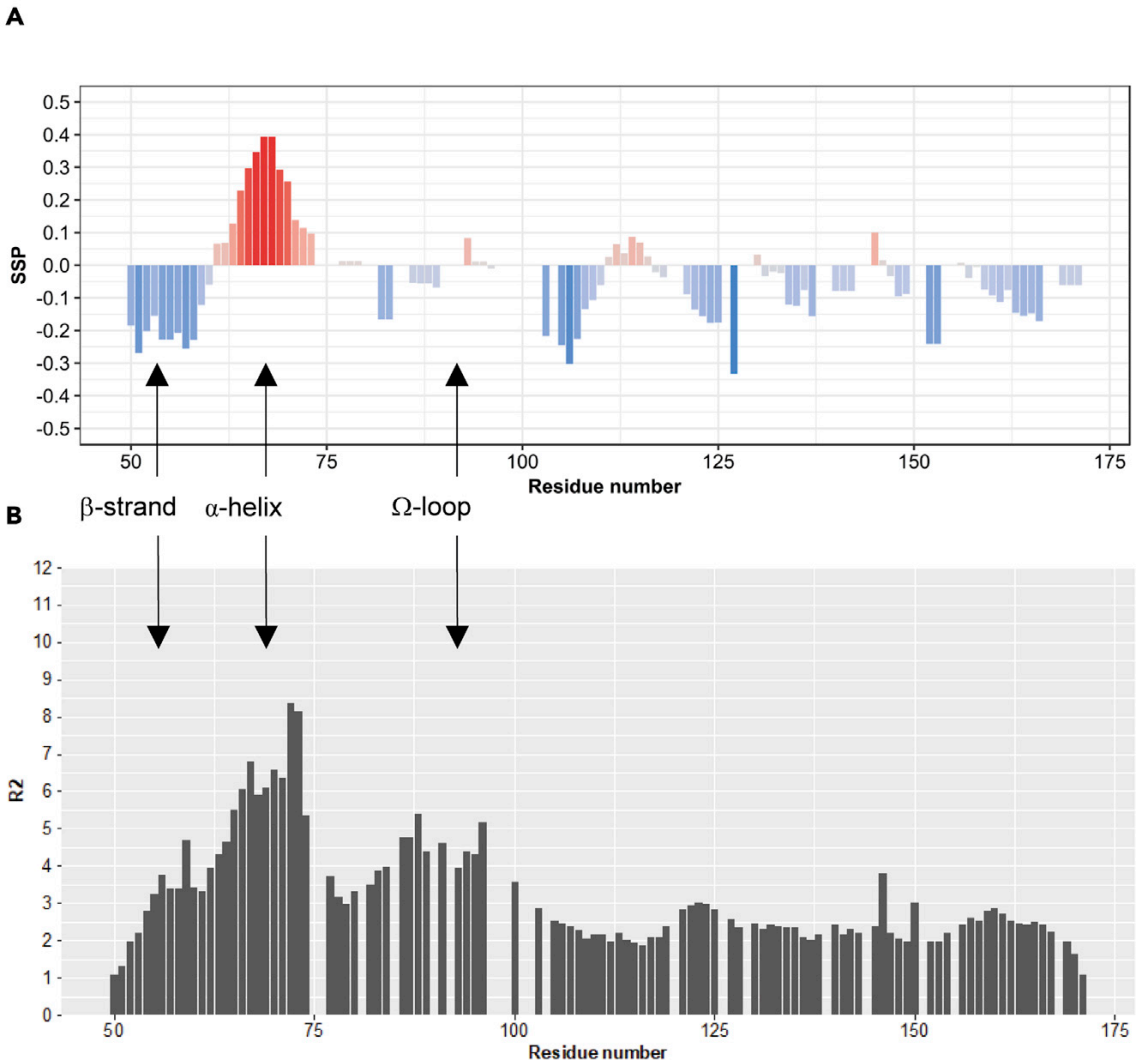
Chemical shift and relaxation data indicate preformation of secondary structure elements in the apo form of YAP (A) SSP score (Marsh et al., 2006) of YAP^wt^ at pH 6 and 298 K. Positive scores indicate a propensity for α-helical structures, whereas β-strands or extended structural elements possess a negative score. SSP scores are not applicable to non-canonical secondary structure elements like the Ω-loop. (B) ^15^N R_2_ Rates of YAP^wt^ at pH 6 and 298 K.

We obtained straightforward evidence that YAP^50-171^ (hereafter referred to as YAP^wt^) possesses all three described secondary structure elements (β-strand, α-helix, and Ω-loop) in the apo state through the combination of chemical shift data, ^15^N relaxation measurements, and site-specific precursor labeling paired with NOE experiments. The secondary structure propensity (SSP) scores (Marsh et al., 2006) derived from the chemical shift data of the ^1^H, ^13^C, and ^15^N and -assignment of YAP (Feichtinger et al., 2018) clearly indicate a propensity for a preformation of an extended structure in the N-terminus of the protein corresponding to the β-strand region and the α-helix in the unbound state (Figure 1A). In particular, the helical region spanning from residue 61-73 in the YAP:TEAD complex shows a strong helical propensity up to an SSP of 0.4 representing the helical population. Residues located in the Ω-loop show no increased SSP scores as this method is not applicable to non-canonical secondary structure elements. This observation of the secondary structure elements preformation is further supported by the utilization of the ^15^N transversal relaxation rates (R_2_) of the backbone amides. The R_2_ values reveal three regions with higher R_2_ relaxation rates that correspond to the three secondary structure elements in the region 50-100 (see Figure 1B). Residues located in the region comprising the α-helix have the highest R_2_ values, as high as 8 s^−1^ at 18.8 T, as well as residues 86-100, which exhibit larger than average relaxation rates. Interestingly, the N-terminus (comprising the β-strand) and the linker region between the α-helix and Ω-loop have significantly lower values.

**Figure 2:**
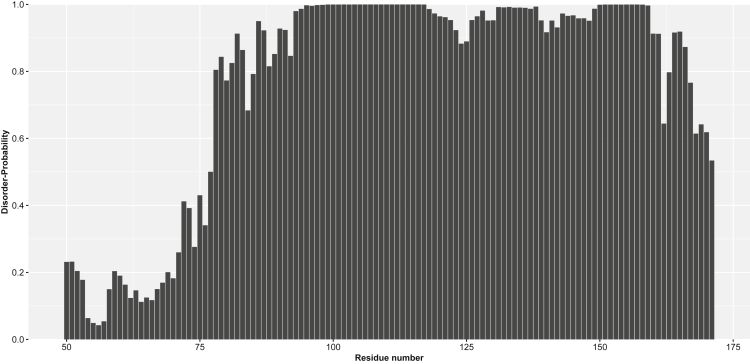
Disorder-probabilities of the YAP^wt^ amino acid sequence calculated via ODiNPred

A comparison of the R_2_ values and SSP scores with the disorder-probabilities (see Figure 2) computed by the deep neural network of ODiNPred (Dass et al., 2020) shows that based on the amino acid sequence of the protein one may expect ordered structures up to residues 72-75 that correspond to the C-terminus of the α-helix. Interestingly, residues 86-100 comprising the Ω-loop region exhibit a significantly increased disorder-probability. The disorder-probabilities’ of residues 101-171 are on average >0.8 and are, therefore, in agreement with the experimental observations.

**Figure 3:**
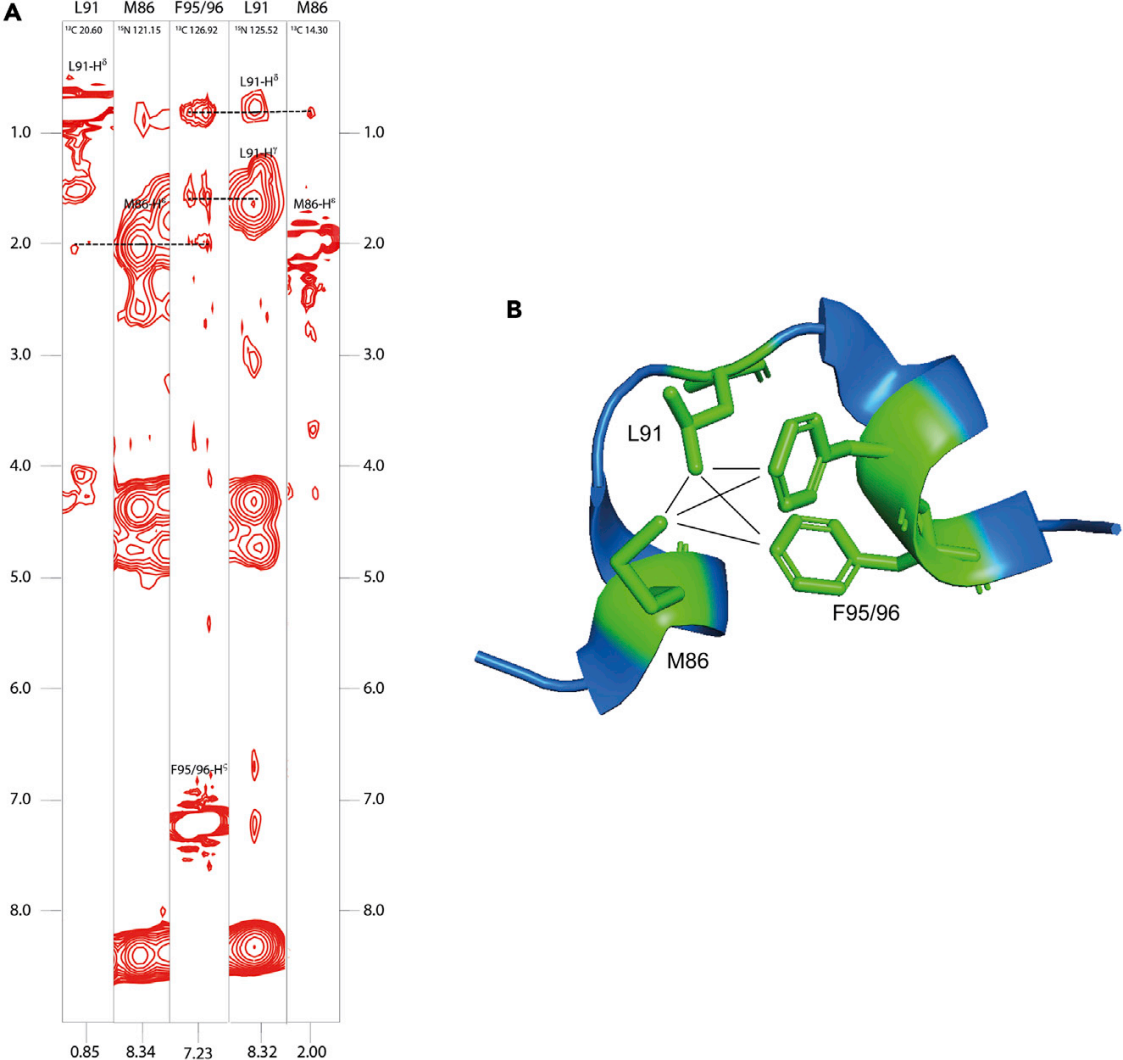
Structural preformation of the Ω-loop in YAP (A) ^13^C-NOESY-HSQC and ^15^N-NOESY-HSQC strips demonstrating the close spatial proximity of the side chains of residues M86, L91, F95/96. (B) Depiction of the NOE contacts observed in the apo form of YAP (dashed lines) in the crystal structure of the YAP:TEAD complex (PDB: 3KYS).

The detection of structural preformation within the Ω-loop region mandated another strategy as the SSP score is not applicable on non-canonical secondary structure elements like an Ω-loop. However, the R_2_ values indicated the prevalence of slower dynamics in this region. Therefore, we used selective side-chain labeling with late metabolic precursors to identify long-range contacts (between residues separated by up to 10 residues in the primary sequence) that indicate the structural preformation of the Ω-loop region (Schörghuber et al., 2018). Based on the information from the crystal structure (Li et al., 2010) and the available biochemical data on the YAP:TEAD interaction (Mesrouze et al., 2017), we decided to specifically label methionine, leucine, and phenylalanine residues. Significant long-range side-chain NOEs between F95/96-H^ς^, M86-H^ε^, and L91-H^δ^ were obtained (see Figure 3).

It was not possible to properly distinguish between F95 and F96 as the mutation of each one of these residues leads to major changes in the ^1^H-^13^C HSQC spectrum of the phenylalanine ^1^H^ς^s (see Figure S1). However, the significant changes provide evidence for the formation of a compact state displaying distinct structural features. As can be seen in Figure 2, residues M86, L91, F95, and F96 show specific long-range NOEs which are in very good agreement with the geometry of the Ω-loop region in the final YAP:TEAD complex (Figure 2B). It can thus be concluded that a structure that resembles the hydrophobic core of the bound Ω-loop region already exists in the apo-state of YAP prior to TEAD binding.

### Secondary structure element preformation is co-dependent

**Figure 4:**
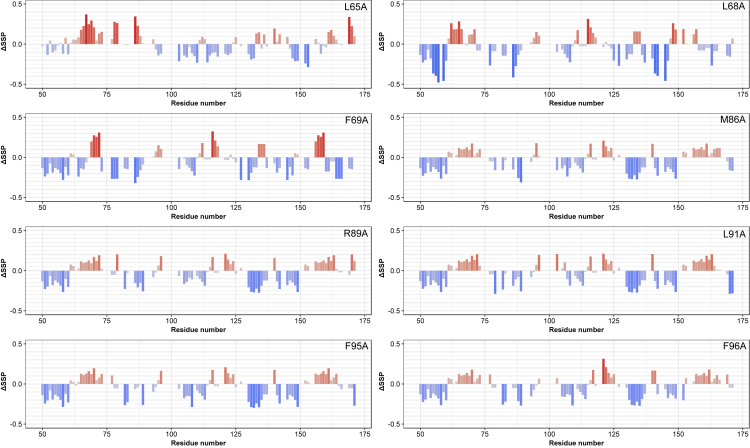
Preformation of secondary structure elements is interdependent ΔSSP (SSP_wt_ – SSP_mutant_) scores demonstrate the relative change in secondary structure propensity upon the introduction of point mutations. Higher values indicate higher deviations from the YAP^wt^ SSP scores.

To provide additional information about this compact state we studied YAP mutants that were previously reported to have a major impact on the formation of the YAP:TEAD complex (Mesrouze et al., 2017). Most importantly, the α-helical propensity is consistently decreased in the different mutants. To visualize the change in secondary structure formation ΔSSP (SSP_wt_ – SSP_mutant_) were calculated (see Figure 4). Although this decrease in the helical propensity is expected for the mutation of residues located in the local LxxLF motif within the α-helix (L65A, L68A, F69A), the decrease of the helical propensity on the introduction of mutations within the Ω-loop region (M86A, R89A, L91A, F95A, F96A) more than 10 residues apart from the α-helix is rather surprising. These findings indicate that a destabilization of the Ω-loop structure through the mutation of crucial hydrophobic residues (M86A, L91A, F95A, F95A) that compose the hydrophobic core of the Ω-loop concurrently destabilizes the α-helical structure and decreases the helical population. In addition, the negatively charged R89 at the N-terminus of the Ω-loop also leads to a decrease of the helical propensity which might be the consequence of the disruption of the electrostatic interaction of this region with the positively charged C-terminus of the α-helix (Mesrouze et al., 2021). Interestingly, mutations that lead to more pronounced changes in the binding affinity of the YAP:TEAD complex (R89A, L91A, F95A, F96A) exhibit higher ΔSSP-values in the α-helical region. In addition, the propensity for an extended structure also decreases in the N-terminal β-strand region for all mutants except for L68A. This might be further supporting the notion that also the N-terminal extended structure that corresponds to β-strand region in the bound state undergoes some change on the introduction of these point mutations. Therefore, one can draw the hypothesis that the preformation of the extended structure at the N-terminus, α-helix, and Ω-loop are co-dependent. These results demand a more detailed investigation of the change in the long-range backbone dynamics within this N-terminal region of YAP^wt^.

### Long-range structural compaction of YAP^wt^

Paramagnetic relaxation enhancement (PRE), which is one of the most relevant experimental approaches to probe for transient long-range contacts in IDPs (Kosen, 1989), was applied to YAP^wt^. The presence of a paramagnetic (1-oxyl-2,2,5,5-tetramethyl-Δ3-pyrroline-3-methyl) methanethio-sulfonat (MTSL) spin label that is introduced at several protein positions leads to an enhancement of the transverse relaxation rates R_2_ depending on the inverse sixth power of the distance (1/r^6^) between the unpaired electron and the observed nucleus of the backbone amide hydrogen. To obtain a sufficient resolution (Silvestre-Ryan et al., 2013) of the whole protein fragment, 12 cysteines were introduced via site-directed mutagenesis at different positions: D60, A71, V80, K90, S103, T119, S127, S138, S149, Q158, S164, 172 (insertion). To determine if these mutations affect the YAP:TEAD interaction, the potency (IC_50_) of the mutants was measured in a TR-FRET assay where one looks for their ability to compete with a Cy5-labelled version of YAP for binding to TEAD (Bokhovchuk et al., 2020b). The IC_50_ of the mutants is similar to the one obtained with YAP^wt^, indicating that the mutations have no significant effect on their ability to bind to TEAD (see Table S1). Intermolecular PRE effects were excluded by control measurements of mixtures (1:1) of ^15^N-labeled YAP^wt^ and ^14^N MTSL-labeled YAP mutants. For this purpose, rates were measured for mixtures containing the ^14^N MTSL-labeled mutants D60C, S103C, and S164C. The evaluation of all of them showed no significant PRE effects at the ^15^N labeled backbone of YAP^wt^. Therefore, intermolecular PRE effects can be excluded.

**Figure 5:**
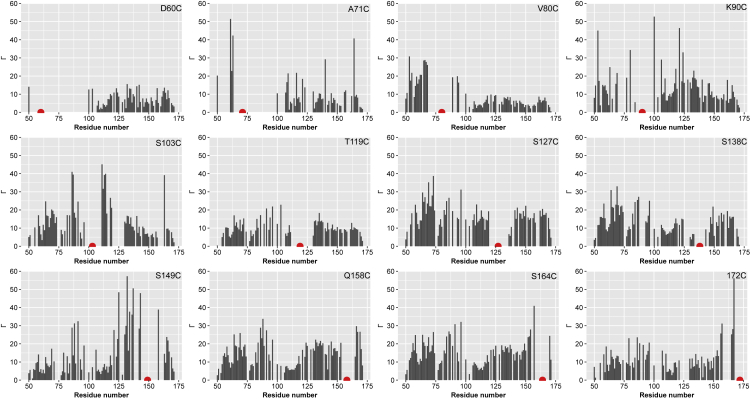
Long-range contacts in apo YAP PRE rates measured by the introduction of MTSL spin labels at 12 different positions indicated by the red dots. High rates indicate spatial proximity between the spin label and the protein residue.

Inspection of Figure 5 provides strong evidence for the formation of compact states in YAP prior to TEAD binding. In particular, when the spin label is placed at positions 60 or 71, almost complete loss of signals is observed for residues of the N-terminus of YAP^wt^ (residues 50-100) owing to efficient paramagnetic relaxation induced by the spin label. In a similar manner, the MTSL spin label at position 90 leads to fast relaxation in the protein region from 65 to 100. The mutation at V80C is located in the loop region connecting the α-helix and Ω-loop in the known crystal structures. In contrast to the labels at position 60 or 71, the spin label attached to position 80 exhibits way fewer long-range backbone interactions than the three aforementioned positions. The data from the first four spin labels suggest a very close spatial proximity of the β-strand region, α-helix, and Ω-loop regions. In particular, the complete loss of the signal over a large region of the protein (∼50 residues) upon the introduction of a spin label in the area of these secondary structure elements supports this hypothesis. Interestingly, higher PRE rates for the first 50 N-terminal residues were also observed for other spin label positions. This observation provides further support for the apparent compaction of the N-terminal segment and even suggests the global compaction of YAP in its apo-state.

**Figure 6:**
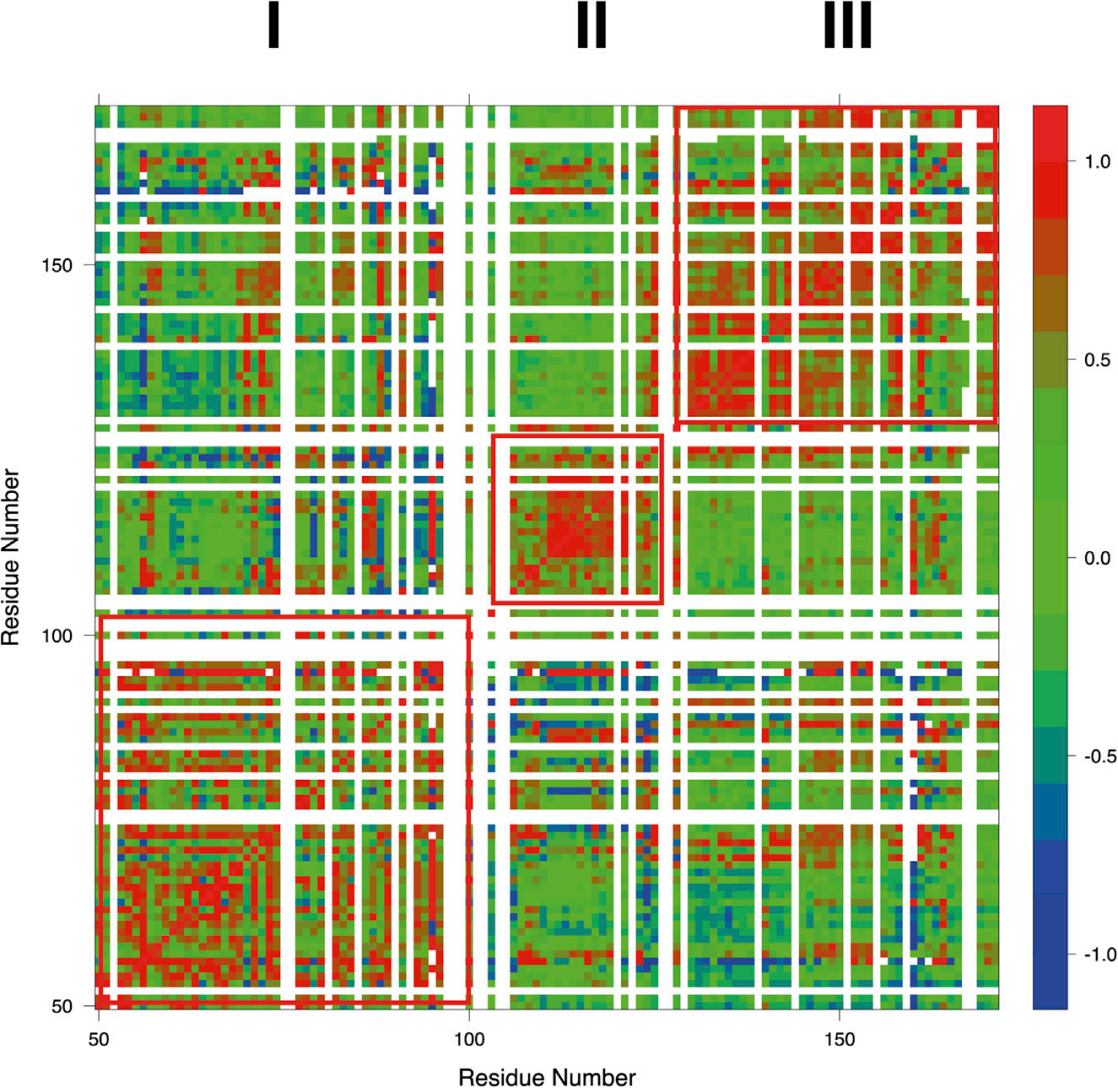
YAP exhibits three structurally correlated regions Correlation analysis of PRE data obtained from 12 different spin labeling positions for YAP on pH 6 and 298 K. Positive correlations (red) indicate structurally correlated protein regions.

As the PRE data provided evidence for a compacted state, we employed the recently introduced correlation matrix analysis of PRE data (see Figure 6). In previous publications, we demonstrated that this approach yields unique insight into the conformational ensemble and concerted structural fluctuations of IDPs (Kurzbach et al., 2017). By applying this methodology to YAP, we are able to identify three regions that may exhibit structural compaction. The first region (I) between residues 50-100 corresponds to the TEAD binding interface identified in the crystal structure. The second positively correlated region around residue 127 (II) corresponds to the 14-3-3 binding site that is phosphorylated for the cytoplasmic retention of YAP. In addition, there seems to be some compaction in the C-terminus of the YAP fragment (III).

**Figure 7:**
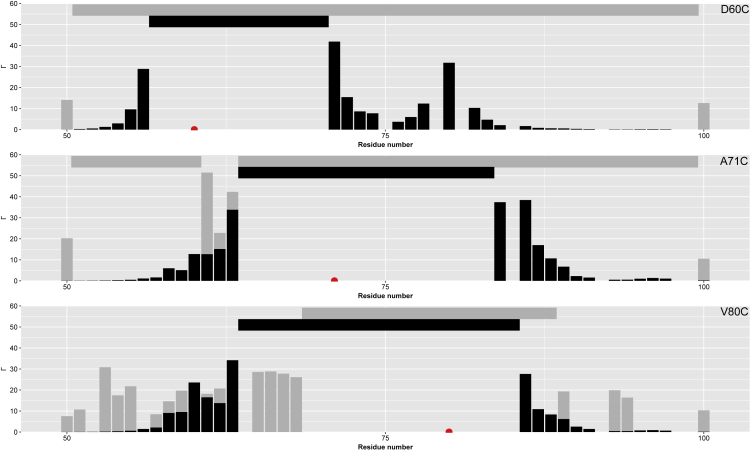
The TEAD-binding domain of YAP exhibits a structural ensemble that contains narrower spatial proximity than the one in the bound state Comparison of experimentally derived (grey) and calculated PRE rates (black). The red dot indicates the position of the MTSL spin label. The horizontal bars indicate residue positions with no PRE value owing to relaxation rates that were not detectable in our experimental setup (grey) or calculated rates >60 s^−1^ that were excluded from the figure as they were not detectable in our experiments (black).

To further compare the degree of “compactness” of the structural ensemble in the apo state in comparison to the bound state, PRE rates were calculated *in silico* from the existing crystal structure (Li et al., 2010). The comparison of experimentally derived PRE rates and PRE rates calculated from the crystal structure suggests that the region between residues 50-100 adopts a structural conformation that may be described as even more compact than the conformation in the bound form (see Figure 7). PRE rates from the crystal structure were calculated under the assumption that in the bound state, the three secondary structure elements of YAP remain relatively rigid and have a well-defined structure. In addition, PRE rates >60 s^−1^ were excluded from the comparison as they were not detectable in our experimental setup. The comparison of the rates obtained for the spin label at position 80 shows that the spatial distance between the loop region and the α-helix increases in the apo state. This indicates a compensation mechanism for the close spatial proximity of the α-helix and Ω-loop in the apo state that sequesters the loop region further away from the α-helix in comparison to the bound state. Only the N-terminal β-strand region seems to be more rigid in the bound state, presumably owing to stabilizing interactions across the interface.

### Yes-associated protein compaction has a kinetic contribution to the YAP:TEAD formation

**Figure 8:**
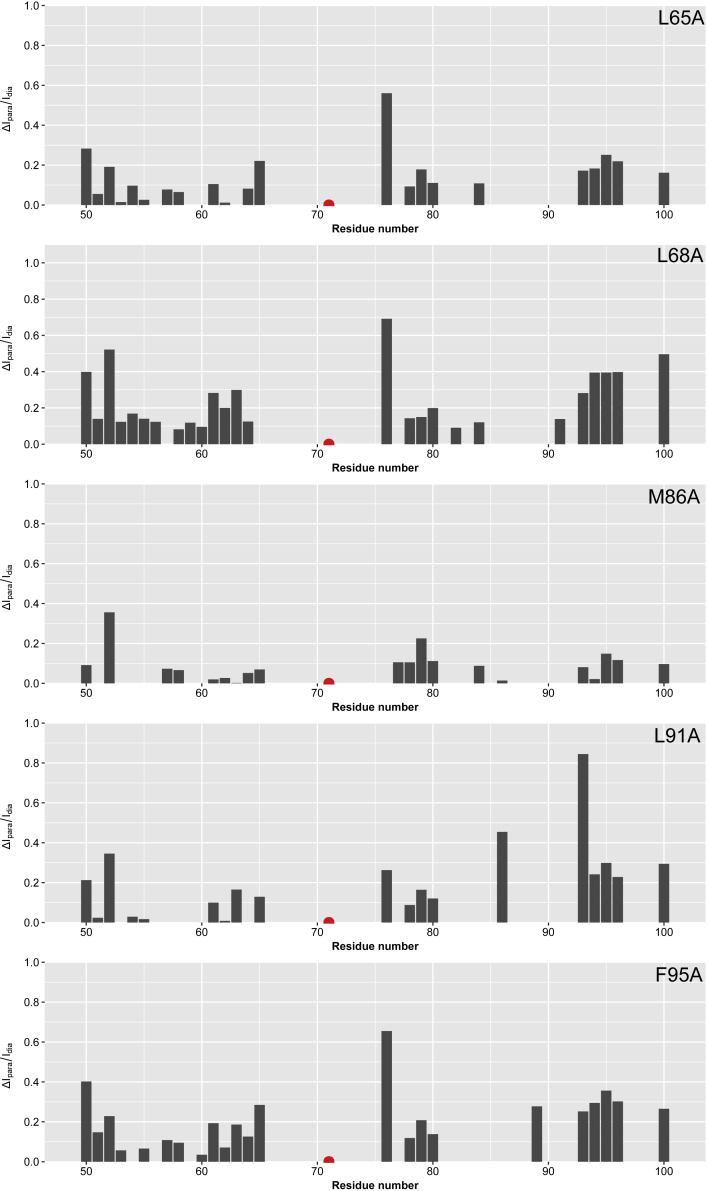
Long-range structural changes on the introduction of mutations The differential between the intensity ratios of the para- and diamagnetic peak intensities of YAP^wt^ with the spin label at A71C and the respective mutations containing the same spin label. Higher values indicate an increase in the spatial proximity between the spin label and the residue position.

To study the impact of the aforementioned mutations on the YAP:TEAD complex formation double mutants were produced that contained the reported mutations and the A71C spin labeling site. Interestingly, the degree of compaction has decreased in all mutated forms of YAP (see Figure 8). Figure 8 shows differential intensity ratios of the para- and diamagnetic peak intensities of YAP^wt^ with the spin label at A71C and the respective mutations also containing the A71C label. PRE differences for residues located in the region 101-171 are insignificant and are thus not shown. Inspection of Figure 8 reveals that the biggest difference in the PRE profiles is observed for the mutation L68A, which is part of the LxxLF motif in the α-helix. Specifically, residues 93-96 located at the C-terminal part of the Ω-loop exhibit a sizeable increase in the intensity ratios indicative of distinct spatial separation between the α-helix and Ω-loop in the mutant. The mutations L65A, M86A, and L91A primarily exhibit changes within the Ω-loop region, while the mutation F95A affects the β-strand and α-helix region in a significant manner. To conclude, the differential PRE data are in very good agreement with the observed reduction of secondary structure formation in the mutant forms and provide additional experimental evidence for the compact apo-state of YAP^wt^.

To further corroborate the expansion of the YAP conformational ensembles, diffusion-ordered spectroscopy (DOSY) measurements of different mutants were performed (see Table 1). To increase the sensitivity for changes in the N-terminal region of YAP, a new construct ranging from residue 50 to 103 was produced and mutated at the respective sites. A comparison of the ^1^H-^15^N HSQC spectra exhibited a nearly perfect overlay of all peaks in the 50-103 construct and YAP^wt^. The truncation, therefore, does not induce any significant changes in the structural ensembles. Inspection of the DOSY values yields a similar picture as the PRE data. The mutations L68A and F95A that lead to higher degrees of decompaction show slower diffusion constants. The mutation of F69A has the strongest effect on the diffusion constant.

**Table 1:** Kinetic effects of different YAP mutations visualized through diffusion coefficients (D)

	Mutation	D (10^−11^ m^2^ s^−1^)
α-helix	wt	13.8 ± 0.1
	L68A	12.1 ± 0.1
	F69A	11.2 ± 0.1
Ω-loop	M86A	13.2 ± 0.1
	L91A	12.88 ± 0.02
	F95A	11.8 ± 0.1

Previous studies have shown that the same mutations change the kinetics of the YAP:TEAD formation and decrease the value of the association rate, k_on_, for the complex formation (Bokhovchuk et al., 2020b). Their Φ-value analysis revealed that the α-helix already exists in the encounter complex preceding complex formation. Interestingly, the Ω-loop mutations induce on average smaller changes of the diffusion constant than the ones in the α-helix, indicating that the α-helix site contributes more to the global compact of YAP^wt^ and may be kinetically favored if YAP in its apo-state is adopting a more compacted and, therefore, faster diffusing state.

In addition to previous publications, our results about the compaction of YAP in its apo form suggest that there is an additional conformational selection step of the YAP:TEAD binding process happening on a nanosecond timescale that could not be detected in previous stopped-flow experiments, but becomes clear through NMR. Thus, our NMR data again demonstrate the existence of a compact apo-state of YAP and its relevance for the formation of the encounter complex with TEAD.

## Discussion

Previous publications dissected and characterized the YAP:TEAD binding site (Chen et al., 2010; Hau et al., 2013; Li et al., 2010; Mesrouze et al., 2017) and provided a first glimpse on the mechanism of protein complex formation (Bokhovchuk et al., 2020b). However, structural information about the apo state of YAP and how putative structural preformation might influence its binding to TEAD has not yet been reported. The observed global compaction of apo YAP results from subtle and transient stabilizing interactions between localized secondary structures. In particular, the α-helical LxxLF (L65 to F69) motif and the Ω-loop region (M86 to F96) are crucial for the stabilization of a hydrophobic core resulting in the transient formation of a dynamic yet compact structure with distinct long-range correlations. Most importantly, these data are in very good agreement with prior mutational and kinetic studies that identified these residues as crucial for the nanomolar affinity between YAP and TEAD (Mesrouze et al., 2017) and, therefore, linking the preformation with the YAP:TEAD binding.

Surprisingly, the comparison of experimental PRE rates with predicted PRE rates obtained from the crystal structure of the YAP:TEAD complex revealed a structural arrangement in the apo form in which the secondary structure elements are already formed and even closer in space than in the bound form. In addition, we demonstrated that the C-terminal region outside the TEAD-binding domain also exhibits significant structural compaction. Interestingly, this region contains additional protein interaction sites (i.e. 14-3-3 binding site), and it seems plausible that these additional compaction “hot-spots” are relevant for YAP’s biological functionality by fine-tuning interaction networks with authentic partners. Transient intramolecular contacts between the individual subdomains in YAP might, therefore, provide a versatile and effective regulatory access control mechanism.

Using a combination of site-directed mutagenesis, ^13^C chemical shifts, PRE measurements, and NMR-diffusion (DOSY) measurements, we could demonstrate that structural preformation and compaction in YAP is driven by the formation of a hydrophobic core established by residues from the previously described α-helix and Ω-loop. This is somewhat surprising as previous studies have shown that there is only a very weak correlation between increased hydrophobicity and structural compaction in IDPs. Marsh and Forman-Kay concluded that usually, the main facilitators of IDP compaction are net charge and proline content (Marsh and Forman-Kay, 2010). YAP, thereby, serves as an example for an IDP whose compaction is also driven by mechanisms reminiscent of folded (globular) proteins and thus questioning the conventional notion of IDPs being largely disordered. Furthermore, the formation of this hydrophobic core in YAP may be an example of the functional misfolding of an IDP to prevent unwanted interactions with non-native partners (Uversky, 2011). Uversky claims that there is chance that IDPs misfold to sequester the preformed elements inside the non-interactive or less-interactive cage as observable in the surprisingly compact apo state of YAP in which the interaction of the α-helix and Ω-loop may prevent their hydrophobic regions from unwanted interactions.

The observed structural compaction of apo YAP also provides a compelling structural explanation for the proposed mechanistic model of the YAP:TEAD complex formation (Bokhovchuk et al., 2020b). According to this study, the α-helix binding interface is formed at an earlier stage of the binding event, which is in good agreement with our data that show that mutations of the LxxLF motif of the α-helix have the biggest impact on the overall compaction of YAP and, thereby, may as well influence the binding kinetics. The initial α-helical preformation serves as a scaffold for the formation and, consequently, co-stabilization of the other two secondary structure elements. The resulting compact substrate allows for a rapid and efficient formation of a protein encounter complex that subsequently readjusts to the final shape. We thus propose that TEAD binding proceeds largely via conformational selection of the preformed compact substate that is happening on a nanosecond timescale and, therefore, was not detectable with previous methods applied to characterize the binding process.

The observation of long-range structural preformation in apo YAP also provides possible starting points for the inhibition of the YAP:TEAD interaction via targeting of the apo state of YAP as this may work with large hydrophobic molecules such as Verteporfin and hexasubstituted dipyrrins (Gibault et al., 2017) that seemingly inhibit the YAP:TEAD complex through YAP binding (Liu-chittenden et al., 2012). Such compounds may preferentially bind to the compact state (providing a characteristic arrangement of electrostatic and hydrophobic moieties) and might interfere with the co-stabilization function of the α-helix and Ω-loop and, hence, disrupt the compact state of YAP.

To conclude, the surprisingly compact state of apo YAP and its relevance for protein complex formation as well as inhibitory ligand binding illustrates the often neglected structural diversity of IDPs and the possibility to tackle these challenging targets by rational, structure-based approaches.

### Limitations of the study

This study investigates the apo form of YAP^wt^ and the effect of mutations on the formation of local and global structured elements. However, the study was performed only with the TEAD-binding domain of YAP ranging from residues 50-171 and, we, therefore, did not analyze possible interactions between that fragment and other domains of YAP. Furthermore, analysis and mutation of some of the proline residues in YAP may allow further insights into the compaction of the apo state.

## STAR★Methods

### Key resources table

**Table:** 

REAGENT or RESOURCE	SOURCE	IDENTIFIER
**Bacterial and virus strains**		

*E. coli* TOP10	Thermo Fisher	Cat#C404010
*E. coli* Tuner™(DE3)	Sigma-Aldrich	Cat#70625

**Chemicals, peptides, and recombinant proteins**		

YAP^50-171^	Produced by authors	N/A
YAP^50-103^	Produced by authors	N/A
DpnI	Thermo Fisher	Cat#ER1701
recA	Thermo Fisher	Cat#RP-4900
^15^N ammonium chloride	Sigma-Aldrich	Cat#299251
^13^C glucose	Cambridge Isotopes	Cat#CLM-1396
Protease Inhibitor Cocktail	Thermo Fisher	Cat#78429
L-Methionin-(*methyl*-^13^C)	Sigma-Aldrich	Cat#299146
Isotopically labeled Phenylalanine precursor	Produced by Group Lichtenecker, University of Vienna	N/A
Isotopically labeled Leucine precursor	Produced by Group Lichtenecker, University of Vienna	N/A
(1-oxyl-2,2,5,5-tetramethyl-Δ3-pyrroline-3-methyl) methanethio-sulfonat (MTSL)	Sigma-Aldrich	Cat#81213

**Critical commercial assays**		

Phusion Flash PCR Master Mix	Thermo Fisher	Cat#F548L

**Oligonucleotides**		

Oligonucleotides for PCR-mediated mutagenesis of various YAP mutants	See Table S2 for a list of oligonucleotides	N/A

**Recombinant DNA**		

pEtM14 vector containing YAP fusion protein with His6-tag and 3 C cleavage site	Produced by authors	N/A

**Software and algorithms**		

NMRPipe	Delaglio et al., 1995	https://www.ibbr.umd.edu/nmrpipe/
CcpNmr	Vranken et al., 2005	https://ccpn.ac.uk/
Sparky	Goddard and Kneller, 2004	https://www.cgl.ucsf.edu/home/sparky/
RStudio	RStudio Team	https://www.rstudio.com/

### Resource availability

#### Lead contact

Further information and requests for resources and reagents should be directed to and will be fulfilled by the lead contact, Michael Feichtinger (michael.feichtinger@univie.ac.at).

#### Materials availability

• A table containing all oligonucleotides (Table S2. Oligonucleotides used) in this work can be found in the supplemental information.
• All chemicals, plasmids, recombinant proteins, and software used are listed in the key resources table.

### Method details

#### Cloning

The sequence coding for YAP residues 50–171 was cloned into a pETM14 vector by Sequence and Ligation Independent Cloning (SLIC) (Scholz et al., 2013). Therefore, the coding sequence of YAP was amplified by polymerase chain reaction (PCR) using primers containing 25 bp overlapping homologies of the target vector at each end. The remaining copies of the initial template vector were removed by a 1-h digest with the restriction enzyme DPNI. An aliquot of approximately 100 ng of the PCR product (coding sequence of YAP 50–171) was mixed in a total volume of 10 μL with approximately 100 ng of the target pETM14 vector and recA; consequently, the mixture was incubated at 37°C for an additional hour. The recombinase recA recombines the PCR products into the target vector due to the overlapping homologous regions. The whole 10 μL were transformed into *E. coli* TOP10 cells and the resulting plasmids were evaluated by sequencing. The final pETM14 vector contained a YAP fusion protein with His6-tag and a 3 C protease cleavage site at the N-terminus.

#### Site-directed mutagenesis

For PCR-mediated mutagenesis, a single PCR step using full plasmid amplification was used (Carey et al., 2013). Hence, 0.2 μL of each 25 bp primer (concentration of 100 μM), 50 ng of plasmid DNA and 20 μL Phusion Flash PCR Master Mix (Thermo Fisher) were combined in a total volume of 40 μL. Then PCR was run for 20 cycles, each consisting of 30 s of denaturation at 96°C and of 2 min of elongation at 72°C. The remaining copies of the initial template vector were removed by a 1-h digest with the restriction enzyme DPNI. Afterwards, 2 μL of each PCR product were transformed into *E. coli* TOP10 cells and the resulting plasmids were evaluated by sequencing.

#### Protein expression and purification

The plasmid, containing the fusion protein with a His6-tag at the N-terminus and a 3C protease cleavage site, was transformed into *E. coli* Tuner™(DE3). Four liters of LB medium, supplemented with Kanamycin, were inoculated with the overnight culture and incubated at 37°C until an OD_600_ of 0.8. The cells were centrifuged and all the pellets were combined and resuspended in one liter of M9 minimal medium containing 1 g/L ^15^N ammonium chloride (Sigma-Aldrich) and 3 g/L ^13^C glucose (Cambridge Isotope) as the sole source of nitrogen and carbon, respectively (Marley et al., 2001). After an additional hour at 37°C, the expression was induced with the addition of 0.8 mM IPTG and cells were incubated for 18 h at 30°C and consecutively harvested by centrifugation and stored at −20°C.

Frozen cell pellets were resuspended in lysis buffer (20 mM Tris, 150 mM NaCl, 30 mM Imidazole, pH 7.8), supplemented with 50 μL of Protease Inhibitor Cocktail (Thermo Scientific), and lysed by sonication on ice. The lysate was clarified by centrifugation and the supernatant was loaded onto a HisTrap FF column and washed with 8 column volumes of wash buffer (20 mM Tris, 150 mM NaCl, 40 mM Imidazole, pH 7.8). After eluting with 15 mL of elution buffer (20 mM Tris, 150 mM NaCl, 300 mM Imidazole, 1 mM EDTA, pH 7.8), the eluate was concentrated to 2 mL and dialyzed overnight against an excess of NMR buffer (20 mM BisTris, 150 mM NaCl, 1 mM EDTA, pH 6.0). Consequently, the His6-tag was removed by 3C protease cleavage overnight at 4°C. NaCl was added to the final concentration of 1 M and the solution was heated to 90°C for 10 min. After centrifugation, the supernatant was loaded onto a HiLoad 16/600 (GE Healthcare) size exclusion column, equilibrated with the NMR buffer. Fractions containing the protein were pooled and concentrated. In the case of the Cysteine mutants, all the buffers were supplemented with 1 mM DTT.

Purified protein, which was >95% pure as assessed by SDS/PAGE (see Figures 2, 3, and 4), was concentrated to approximately 0.5 mM in the NMR buffer containing 10% D_2_O. Owing to the lack of absorbance at 280 nm, the protein concentration was determined by the Pierce BCA Protein Assay (Thermo Scientific).

#### MTSL tagging

The MTSL tagging was initiated combining 1 mL of protein solution with 1 mL of MTSL reaction buffer (100 mM NaP, 1 mM EDTA, pH 8.0) and by incubating it at RT for 15 min after the addition of a 10-fold excess (in comparison to the protein concentration) of DTT. This ensures that all the Cysteines are reduced for the MTSL-tagging procedure. Afterwards, DTT was removed by the PD-10 desalting column and the protein solution was eluted in 3.3 mL of MTSL reaction buffer. From a 50 mM DMSO stock solution, two times excess of MTSL was added to the protein solution. Consequently, the protein solution containing MTSL was incubated for 3 h at 30°C with agitation. Finally, the free MTSL was removed and the sample concentrated by exchange with the NMR Buffer via a Amicon Centricon MWCO 3 kDa. For the measurement of the diamagnetic control experiments, the free electron of the MTSL tag was reduced by a two-fold excess of ascorbic acid.

#### Precursor labeling

The precursor labeling was done by the addition of 1 g/L of ^13^C^ε^-methionine (Sigma-Aldrich) and 10 mg/mL of the isotopically labeled phenylalanine and leucine precursors to the M9 minimal medium used for protein expression (Klopp et al., 2018; Lichtenecker et al., 2013a, 2013b).

#### TR-FRET

Biotinylated N-Avitagged-TEAD4^217–434^ (1 nM) and LANCE Eu-W1024 Streptavidin (0.5 nM, PerkinElmer) were pre-incubated for 1 h at room temperature in 50 mM HEPES pH 7.4, 100 mM KCl, 0.05% (v/v) Tween-20, 0.25 mM TCEP, 1 mM, and 0.05% (w/v) BSA. Different YAP mutants (20 nM) and serial dilutions of them were added and incubated in white 384-well plates (Greiner Bio-One International) for 1 h at room temperature. DMSO was present at 2% in the assay. The solubility of the peptides in assay buffer was measured by dynamic light scattering with a Dyna Protdevice (Wyatt technology Corp.). The fluorescence in the TR- FRET assay was measured with a Genios Pro reader (Tecan) (50 μs delay between excitation and fluores- cence, 75 μs integration time, excitation wavelength 340 nm, emission wavelengths 620 and 665 nm). Data analyses were carried out using the TR-FRET 665/620 nm emission ratio. The IC_50_ values were obtained by nonlinear regression analysis with GraphPad Prism (GraphPad Software) (Bokhovchuk et al., 2020a).

#### NMR spectroscopy

NMR experiments were performed at 298 K using a Bruker Avance III 800 MHz, Bruker Neo 600 MHz, and Bruker Neo 500 MHz spectrometer. Data were processed using NMRPipe (Delaglio et al., 1995) and Bruker TopSpin. Spectra were analyzed using CcpNmr (Vranken et al., 2005) and Sparky (Goddard and Kneller, 2004). Further data analysis and plots were performed and created, respectively, with RStudio (RStudio Team, 2017).

### Quantification and statistical analysis

The IC_50_ values were obtained by nonlinear regression analysis with GraphPad Prism (Table S1).

## Data Availability

• All data reported in this paper will be shared by the lead contact upon request. • The data does not report original code. • Any additional information required to reanalyze the data reported in this paper is available from the lead contact upon request.

## Footnotes

Supplemental information can be found online at https://doi.org/10.1016/j.isci.2022.104099.

## References

[bib1] Bokhovchuk F., Mesrouze Y., Delaunay C., Martin T., Villard F., Meyerhofer M., Fontana P., Zimmermann C., Erdmann D., Furet P. (2020). Identification of FAM181A and FAM181B as new interactors with the TEAD transcription factors. Protein Sci..

[bib2] Bokhovchuk F., Mesrouze Y., Meyerhofer M., Zimmermann C., Fontana P., Erdmann D., Jemth P., Chène P. (2020). An early association between the α-helix of the TEAD binding domain of YAP and TEAD drives the formation of the YAP:TEAD complex. Biochemistry.

[bib3] Calses P.C., Crawford J.J., Lill J.R., Dey A. (2019). Hippo pathway in cancer: aberrant regulation and therapeutic opportunities. Trends Cancer.

[bib4] Carey M.F., Peterson C.L., Smale S.T. (2013). PCR-mediated site-directed mutagenesis. Cold Spring Harb. Protoc..

[bib5] Chen L., Chan S.W., Zhang X., Walsh M., Lim C.J. (2010). Structural basis of YAP recognition by TEAD4 in the Hippo pathway structural basis of YAP recognition by TEAD4 in the Hippo pathway. Genes Dev..

[bib6] Crawford J.J., Bronner S.M., Zbieg J.R. (2018). Hippo pathway inhibition by blocking the YAP/TAZ–TEAD interface: a patent review. Expert Opin. Ther. Pat..

[bib7] Dass R., Mulder F.A.A., Nielsen J.T. (2020). ODiNPred: comprehensive prediction of protein order and disorder. Sci. Rep..

[bib8] Delaglio F., Grzesiek S., Vuister G.W., Zhu G., Pfeifer J., Bax A. (1995). NMRPipe: a multidimensional spectral processing system based on UNIX pipes. J. Biomol. NMR.

[bib9] Feichtinger M., Sára T., Platzer G., Mateos B., Bokhovchuk F., Chène P., Konrat R. (2018). 1H, 13C, 15N resonance assignment of human YAP 50–171 fragment. Biomol. NMR Assign..

[bib10] Gibault F., Bailly F., Corvaisier M., Coevoet M., Huet G., Melnyk P., Cotelle P. (2017). Molecular features of the YAP inhibitor Verteporfin: synthesis of hexasubstituted dipyrrins as potential inhibitors of YAP/TAZ, the downstream effectors of the Hippo pathway. ChemMedChem.

[bib11] Goddard T.D., Kneller D.G. (2004).

[bib12] Hau J.C., Erdmann D., Mesrouze Y., Furet P., Fontana P., Zimmermann C., Schmelzle T., Hofmann F., Chène P. (2013). The TEAD4-YAP/TAZ protein-protein interaction: expected similarities and unexpected differences. ChemBioChem.

[bib13] Holden J.K., Cunningham C.N. (2018). Targeting the Hippo pathway and cancer through the TEAD family of transcription factors. Cancers (Basel).

[bib14] Klopp J., Winterhalter A., Gébleux R., Scherer-becker D., Ostermeier C., Alvar Gossert D. (2018). Cost-effective large-scale expression of proteins for NMR studies. J. Biomol. NMR.

[bib15] Kosen P.A. (1989). Spin labeling of proteins. Methods Enzymol..

[bib16] Kurzbach D., Beier A., Vanas A., Flamm A.G., Platzer G., Schwarz T.C., Konrat R. (2017). NMR probing and visualization of correlated structural fluctuations in intrinsically disordered proteins. Phys. Chem. Chem. Phys..

[bib17] Li Z., Zhao B., Wang P., Chen F., Dong Z., Yang H. (2010). Structural insights into the YAP and TEAD complex service Structural insights into the YAP and TEAD complex. Genes Dev..

[bib18] Lichtenecker R.J., Coudevylle N., Konrat R., Schmid W. (2013). Selective Isotope labelling of leucine residues by using α-ketoacid precursor compounds. ChemBioChem.

[bib19] Lichtenecker R.J., Weinhäupl K., Schmid W., Konrat R. (2013). α-Ketoacids as precursors for phenylalanine and tyrosine labelling in cell-based protein overexpression. J. Biomol. NMR.

[bib20] Liu-chittenden Y., Huang B., Shim J.S., Chen Q., Lee S., Anders R.A., Liu J.O., Pan D. (2012). Genetic and pharmacological disruption of the TEAD–YAP complex suppresses the oncogenic activity of YAP. Genes Dev..

[bib21] Liu A.M., Wong K.F., Jiang X., Qiao Y., Luk J.M. (2012). Regulators of mammalian Hippo pathway in cancer. Biochim. Biophys. Acta.

[bib22] Ma S., Meng Z., Chen R., Guan K.-L. (2019). The Hippo pathway: biology and pathophysiology. Annu. Rev. Biochem..

[bib23] Ma Y., Yang Y., Wang F., Wei Q., Qin H. (2015). Hippo-YAP signaling pathway: a new paradigm for cancer therapy. Int. J. Cancer.

[bib24] Marley J., Lu M., Bracken C. (2001). A method for efficient isotopic labeling of recombinant proteins. J. Biomol. NMR.

[bib25] Marsh J.A., Singh V.K., Jia Z., Forman-Kay J.D. (2006). Sensitivity of secondary structure propensities to sequence differences between alpha- and gamma-synuclein: implications for fibrillation. Protein Sci..

[bib26] Marsh J.A., Forman-Kay J.D. (2010). Sequence determinants of compaction in intrinsically disordered proteins. Biophys. J..

[bib27] Mesrouze Y., Bokhovchuk F., Meyerhofer M., Fontana P., Zimmermann C., Martin T., Delaunay C., Erdmann D., Schmelzle T., Chène P. (2017). Dissection of the interaction between the intrinsically disordered YAP protein and the transcription factor TEAD. Elife.

[bib28] Mesrouze Y., Bokhovchuk F., Meyerhofer M., Zimmermann C., Fontana P., Erdmann D., Chène P. (2021). Study of the TEAD-binding domain of the YAP protein from animal species. Protein Sci..

[bib29] Moya I.M., Halder G. (2019). Hippo–YAP/TAZ signalling in organ regeneration and regenerative medicine. Nat. Rev. Mol. Cell Biol..

[bib30] Pobbati A.V., Han X., Hung A.W., Weiguang S., Huda N., Chen G.Y., Kang C.B., Chia C.S.B., Luo X., Hong W., Poulsen A. (2015). Targeting the central pocket in human transcription factor TEAD as a potential cancer therapeutic strategy. Structure.

[bib31] Pobbati A.V., Hong W. (2013). Emerging roles of TEAD transcription factors and its coactivators in cancers. Cancer Biol. Ther..

[bib32] RStudio Team (2017). http://http//www.rstudio.com.

[bib33] Santucci M., Vignudelli T., Ferrari S., Mor M., Scalvini L., Bolognesi M.L., Uliassi E., Costi M.P. (2015). The Hippo pathway and YAP/TAZ-TEAD protein-protein interaction as targets for regenerative medicine and cancer treatment. J. Med. Chem..

[bib34] Scholz J., Besir H., Strasser C., Suppmann S. (2013). A new method to customize protein expression vectors for fast, efficient and background free parallel cloning. BMC Biotechnol..

[bib35] Schörghuber J., Geist L., Platzer G., Feichtinger M., Bisaccia M., Scheibelberger L., Weber F., Konrat R., Lichtenecker R.J. (2018). Late metabolic precursors for selective aromatic residue labeling. J. Biomol. NMR.

[bib36] Silvestre-Ryan J., Bertoncini C.W., Fenwick R.B., Esteban-Martin S., Salvatella X. (2013). Average conformations determined from PRE data provide high-resolution maps of transient tertiary interactions in disordered proteins. Biophys. J..

[bib37] Tian W., Yu J., Tomchick D.R., Pan D., Luo X. (2010). Structural and functional analysis of the YAP-binding domain of human TEAD2. Proc. Natl. Acad. Sci. U S A.

[bib38] Tremblay A.M., Camargo F.D. (2012). Hippo signaling in mammalian stem cells. Semin. Cell Dev. Biol..

[bib39] Uversky V.N. (2011). Intrinsically disordered proteins may escape unwanted interactions via functional misfolding. Biochim. Biophys. Acta.

[bib40] Vassilev A., Kaneko K.J., Shu H., Zhao Y., Depamphilis M.L. (2001). YAP65 , a Src/Yes-associated protein localized in the cytoplasm TEAD/TEF transcription factors utilize the activation domain of YAP65 , a Src/Yes-associated protein localized in the cytoplasm. Genes Dev..

[bib41] Vranken W.F., Boucher W., Stevens T.J., Fogh R.H., Pajon A., Llinas M., Ulrich E.L., Markley J.L., Ionides J., Laue E.D. (2005). The CCPN data model for NMR spectroscopy: development of a software pipeline. Proteins.

[bib42] Wang Y., Yu A., Yu F.X. (2017). The Hippo pathway in tissue homeostasis and regeneration. Protein Cell.

[bib43] Ye S., Eisinger-Mathason T.S.K. (2016). Targeting the Hippo pathway: clinical implications and therapeutics. Pharmacol. Res..

[bib44] Zhao B., Zhao B., Wei X., Wei X., Li W., Li W., Udan R.S., Udan R.S., Yang Q., Yang Q. (2007). Inactivation of YAP oncoprotein by the Hippo pathway is involved in cell contact inhibition and tissue growth control. Genes Dev..

[bib45] Zhao B., Tumaneng K., Guan K.L. (2011). The Hippo pathway in organ size control, tissue regeneration and stem cell self-renewal. Nat. Cell Biol..

