# Supplementary material for: Long-range structural preformation in yes-associated protein precedes encounter complex formation with TEAD
Source: iScience. 2022 Mar 17;25(4):104099. doi: 10.1016/j.isci.2022.104099 (PMC8976148; doi:10.1016/j.isci.2022.104099)
Supplement: Document S1. Figure S1, Tables S1, and S2 [file mmc1.pdf]

iScience, Volume *25*

Supplemental information

Long-range structural preformation in yes-associated protein precedes encounter complex formation with TEAD

Michael Feichtinger, Andreas Beier, Mario Migotti, Matthias Schmid, Fedir Bokhovchuk, Patrick Chène, and Robert Konrat

Supplemental Information

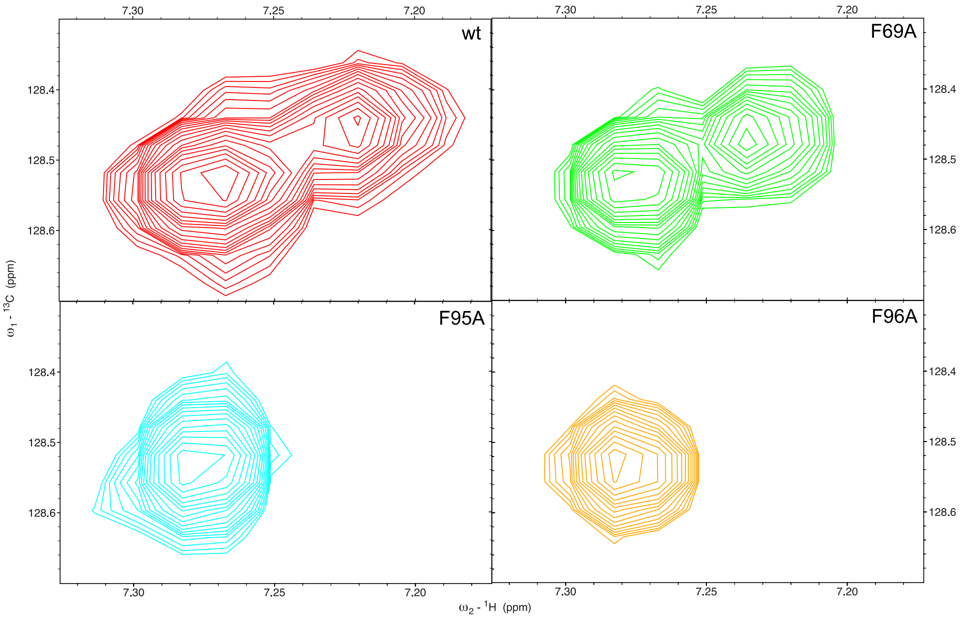

Fig. S1: 1H-13C HSQCs of labeled phenylalanine side-chains

YAP Mutations	Av IC_{50} (nM)	SD
D60C	12	2
A71C	9	2
V80C	4	1
K90C	12	1
S103C	2.9	0.4
A112C	3	1
T119C	5	1
S137C	5.3	0.4
S138C	4	1
S149C	2.6	0.4
Q158C	2.9	0.4
S164C	1.9	0.4
172C (insertion)	7	1
wt	11	1

Table S1: Potency of PRE mutants. The potency of the different mutant was measured in a TR-FRET assay ($n \geq 2$).

	Sequences of the oligonucleotides (5’-3’)	
D60C	GATCGTGCACGTCCGCGGGTGCTCGGAGACCGACCTGGAGGCG	CGCCTCCAGGTCGGTCTCCGAGCACCCGCGGACGTGCACGATC
A71C	CCTGGAGGCGCTCTTCAACTGCGTCATGAACCCCAAGACGGC	GCCGTCTTGGGGTTCATGACGCAGTTGAAGAGCGCCTCCAGG
V80C	GAACCCCAAGACGGCCAACTGTCCCCAGACCGTGCCCATGAGGC	GCCTCATGGGCACGGTCTGGGGACAGTTGGCCGTCTTGGGGTTC
K90C	CCGTGCCCATGAGGCTCCGGTGTCTGCCCGACTCCTTCTTCAAGC	GCTTGAAGAAGGAGTCGGGCAGACACCGGAGCCTCATGGGCACGG
S103C	CCGCCGGAGCCCAAATGCCACTCCCGACAGGCCAG	CTGGCCTGTCGGGAGTGGCATTTGGGCTCCGGCGG
A112C	CCCGACAGGCCAGTACTGATTGTGGCACTGCAGGAGCCCTGAC	GTCAGGGCTCCTGCAGTGCCACAATCAGTACTGGCCTGTCGGG
T119C	GGCACTGCAGGAGCCCTGTGTCCACAGCATGTTCGAGCTC	GAGCTCGAACATGCTGTGGACACAGGGCTCCTGCAGTGCC
S127C	CCACAGCATGTTCGAGCTCATTGCTCTCCAGCTTCTCTGCAGTTG	CAACTGCAGAGAAGCTGGAGAGCAATGAGCTCGAACATGCTGTGG
S138C	GCAGTTGGGAGCTGTTTGTCCTGGGACACTGACCCCCACTGG	CCAGTGGGGGTCAGTGTCCCAGGACAAACAGCTCCCAACTGC
S149C	GTGGGTGTAGCTGCTGGGCCACAGACTACTCCAGTGGGGGTC	GACCCCCACTGGAGTAGTCTGTGGCCCAGCAGCTACACCCAC
Q158C	CCCAGCAGCTACACCCACAGCTTGTCATCTTCGACAGTCTTC	GAAGACTGTCGAAGATGACAAGCTGTGGGTGTAGCTGCTGGG
S164C	GCTCAGCATCTTCGACAGTCTTGTTTTGAGATACCTGATGATG	CATCATCAGGTATCTCAAAACAAGACTGTCGAAGATGCTGAGC
172C	GAGATACCTGATGATGTATGTTAACGCCATTAACCTGATGTTCTGG	CCAGAACATCAGGTTAATGGCGTTAACATACATCATCAGGTATCTC
L65A	CGGGGACTCGGAGACCGACGCGGAGGCGCTCTTCAACGCCG	CGGCGTTGAAGAGCGCCTCCGCGTCGGTCTCCGAGTCCCCG
L68A	GGAGACCGACCTGGAGGCGGCCTTCAACGCCGTCATGAACC	GGTTCATGACGGCGTTGAAGGCCGCCTCCAGGTCGGTCTCC
F69A	CCGACCTGGAGGCGCTCGCCAACGCCGTCATGAACCCC	GGGGTTCATGACGGCGTTGGCGAGCGCCTCCAGGTCGG
M86A	GTGCCCCAGACCGTGCCCGCGAGGCTCCGGAAGCTGCCC	GGGCAGCTTCCGGAGCCTCGCGGGCACGGTCTGGGGCAC
R89A	GACCGTGCCCATGAGGCTCGCGAAGCTGCCCGACTCCTTC	GAAGGAGTCGGGCAGCTTCGCGAGCCTCATGGGCACGGTC

L91A	GCCCATGAGGCTCCGGAAGGCCCCCGACTCCTTCTTCAAGC	GCTTGAAGAAGGAGTCGGGGGCCTTCCGGAGCCTCATGGGC
F95A	GGAAGCTGCCCGACTCCGCCTTCAAGCCGCCGGAGCCC	GGGCTCCGGCGGCTTGAAGGCGGAGTCGGGCAGCTTCC
F96A	GGAAGCTGCCCGACTCCTTCGCCAAGCCGCCGGAGCCCAAATCCC	GGGATTTGGGCTCCGGCGGCTTGGCGAAGGAGTCGGGCAGCTTCC

Table S2: Oligonucleotides used to produce the different YAP mutants via PCR-mediated mutagenesis. The first column denotes the introduced mutation with the two respective primers in the second and third column, Related to STAR Methods.
